# PsnWRKY70 Negatively Regulates NaHCO_3_ Tolerance in *Populus*

**DOI:** 10.3390/ijms232113086

**Published:** 2022-10-28

**Authors:** Wei Wang, Xiang-Dong Bai, Kun Chen, Xiao-Yue Zhang, Chen-Rui Gu, Jing Jiang, Chuan-Ping Yang, Gui-Feng Liu

**Affiliations:** State Key Laboratory of Tree Genetics and Breeding, Northeast Forestry University, 26 Hexing Road, Harbin 150040, China

**Keywords:** poplar, *PsnWRKY70*, transcription factor, NaHCO_3_ stress, gene expression

## Abstract

Poplar is an important afforestation and ornamental tree species in Northeast China. The distribution area of saline-alkali land is approximately 765 hm^2^ in Northeast China. The breeding of saline-alkali-resistant transgenic trees could be an effective method of afforestation in saline-alkali land. WRKY transcription factors play a crucial role in abiotic stress. In this study, we analyzed the genetic stability of the two-year-old *PsnWRKY70* transgenic poplars. The results showed that *PsnWRKY70* of transgenic poplars had been expressed stably and normally at the mRNA level. The gene interference expression (RE) lines had no significant effect on the growth of *PsnWRKY70* under NaHCO_3_ stress, and the alkali damage index of RE lines was significantly lower than that of WT and overexpression (OE) lines at day 15 under NaHCO_3_ stress. POD activity was significantly higher in RE lines than in WT. The MDA content of the RE line was lower than that of the WT line. Transcriptome analysis showed that RE lines up-regulated genes enriched in cell wall organization or biogenesis pathway-related genes such as *EXPA8*, *EXPA4*, *EXPA3*, *EXPA1*, *EXPB3*, *EXP10*, *PME53*, *PME34*, *PME36*, *XTH9*, *XTH6*, *XTH23*, *CESA1*, *CESA3*, *CES9*; *FLA11*, *FLA16* and *FLA7* genes. These genes play an important role in NaHCO_3_ stress. Our study showed that the interference expression of the *PsnWRKY70* gene can enhance the tolerance of NaHCO_3_ in poplar.

## 1. Introduction

Plant growth and yield can be severely affected by various biotic and abiotic stresses such as high salinity, drought and extreme temperatures [1,2]. Plants have evolved complex regulatory networks in response to abiotic stress [3]. Transcription factor TFs often act as molecular switches in signaling networks [4]. The WRKY family is one of the largest transcription factor families (TFs) in plants and plays an important role in biotic and abiotic stress by regulating the expression of stress-responsive genes to increase plant adaptation and tolerance of environmental stress [2,5,6,7,8].

The roles of WRKYs in regulating abiotic stresses such as drought and salt stress have been reported [2,5,6,7,8]. *GmWRKY54* positively regulates soybean drought tolerance by activating genes in abscisic acid and Ca2^+^ signaling pathways in soybean (*Glycine max, Wm82*) [9]. In *Arabidopsis*, *WRKY46*, *WRKY54* and *WRKY70* are involved in BR-regulated plant growth and drought response, as *wrky46 wrky54 wrky70* triple mutants are defective in BR-regulated growth and are more tolerant to drought stress [10]. Overexpression of the transcription factor *SlWRKY28* enhances the tolerance of poplar (*Populus davidiana × P. bolleana*) to alkaline stress [11]. In *Arabidopsis*, *GhWRKY1-like* acts as a positive regulator of drought tolerance and promotes ABA biosynthesis by directly interacting with the promoters of *AtNCED2*, *AtNCED5*, *AtNCED6* and *AtNCED9* [12]. *WRKY70* protein belongs to the class III subfamily of the WRKY transcription factor superfamily and plays a key role in both biotic and abiotic stress regulatory networks in plants [13,14]. *MfWRKY70* of *Myrothamnus flabellifolia* as a positive regulator of the abiotic stress response is a potential gene for improving plant drought and salt tolerance [15]. *WRKY70* and *WRKY54* regulate osmotic stress tolerance by regulating stomatal pore size in *Arabidopsis* [14]. A previous study identified 104 PtWRKY genes in poplar [16]. Overexpression of *PtrWRKY19* in transgenic poplars resulted in a significant increase in pith diameter and a decrease in the expression level of lignin biosynthesis genes [17]. Overexpression of *PtRWRKY40* in transgenic poplar can reduce the expression of SA related genes (*PR1.1*, *PR2.1*, *PR5.9*, *CPR5* and *SID2*) and jasmonic acid (JA) related gene *JAZ8*, making it more sensitive to *D. gregaria* infection [18]. A salicylic acid induced *PtrWRKY73* was found in (*Populus trichocarpa*). Overexpression of *PtrWRKY73* in *Arabidopsis* improved resistance to biotrophic pathogens but decreased resistance to necrotrophic pathogens [19]. In poplar *Populus trichocarpa*, *PtrWRKY75* promotes salicylic acid biosynthesis by activating the expression of the downstream PAL gene, reducing stomatal pore size and resisting drought stress with increasing plant water use efficiency [3]. *PagWRKY75* can reduce the scavenging ability of reactive oxygen species and the accumulation of proline under stress and increase the water loss rate of leaves, thereby increasing the tolerance of plants to salt and osmosis [20]. *PyWRKY75* significantly enhanced the uptake and accumulation of CD and the protective effects of antioxidant enzymes (POD, SOD, CAT and APX), non-enzymatic antioxidants (ASA and GSH) and osmotic adjustment substances (soluble sugar), thereby enhancing the high tolerance of poplar to CD stress [21]. The overexpression of C2H2 type II WRKY transcription factor *PeWRKY31* from *Populus tomentosa* enhanced the salt tolerance of transgenic tobacco [22]. However, research on *PsnWRKY70* regulating biotic stress and abiotic stress response is scant.

Poplar is an important afforestation and ornamental tree species in Northeast China which has an area of 765 hm^2^ saline-alkali land. The breeding of saline-alkali-resistant transgenic trees with genetic engineering could be an effective way to utilize saline-alkali land for afforestation. Our previous study showed that the obtained RE lines of *PsnWRKY70* transgenic poplar have significantly improved salt tolerance [23]. The objective of this study is to further investigate the possibility of these transgenic lines growing in saline-alkali land. We tested the transgenic *PsnWRKY70* poplar lines under treatment NaHCO_3_. The gene expressions of related genes were identified by transcriptome. Alkali damage index, growth and physiological parameters were recorded to evaluate the alkali tolerance of transgenic lines. Our results showed that the interference expression of the *PsnWRKY70* gene could significantly improve the tolerance of NaHCO_3_ in poplar. Some saline-alkali resistant genes regulated by *PsnWRKY70* were discovered by transcriptome. This study provides a reference for the molecular design and breeding of saline-alkali resistance in saline-alkali resistant poplar and other trees.

## 2. Results

### 2.1. PCR Validation of PsnWRKY70 Transgenic Poplar

**Figure 1:**
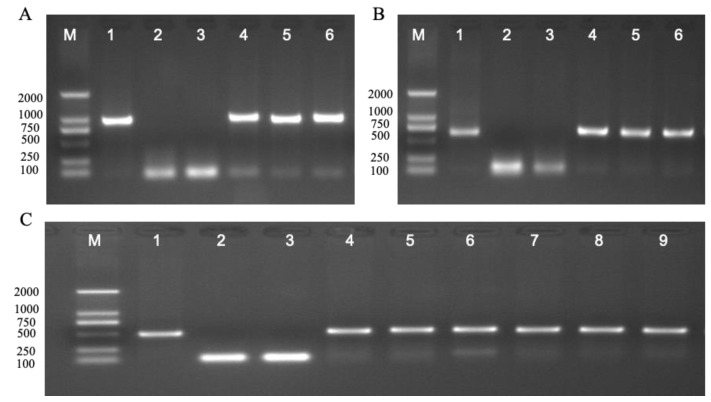
PCR analysis of the transgenic lines and WT lines. (**A**) Specific primers *PsnWRKY70*-F and *PsnWRKY70*-R for amplifying the full length of *PsnWRKY70* ORF, overexpression positive plasmid (lane 1), WT lines DNA (lane 2), water (lane 3), OE1 (lane 4), OE2 (lane 5), OE3 (lane 6). (**B**) *PsnWRKY70* interference specific primers pRS300-F/pRS300-R, l*PsnWRKY70* interference expression positive plasmid (lane 1), WT lines DNA (lane 2), water (lane 3), RE1 (lane 4), RE2 (lane 5), RE3 (lane 6). (**C**) Primers nptII-F and nptII-R, DL2000 DNA marker (M), *PsnWRKY70* overexpression positive plasmid (lane 1), WT (lane 2), water (lane 3), OE1 (lane 4), OE2 (lane 5), OE3 (lane 6), RE1 (lane 7), RE2 (lane 8), RE3 (lane 9).

PCR validation was performed on two-year-old transgenic overexpression OE1-OE3 lines. All three overexpression lines of OE1-OE3 amplified clear specific bands (Figure 1A). Similarly, all RE1-RE3 lines amplified clear specific bands (Figure 1B). Specific primers of kanamycin resistance gene nptII were used for the validation. Figure 1C showed that clear specific bands were amplified in OE and RE lines. This indicates that the genes of two-year-old transgenic lines propagated from cutting were stable.

**Figure 2:**
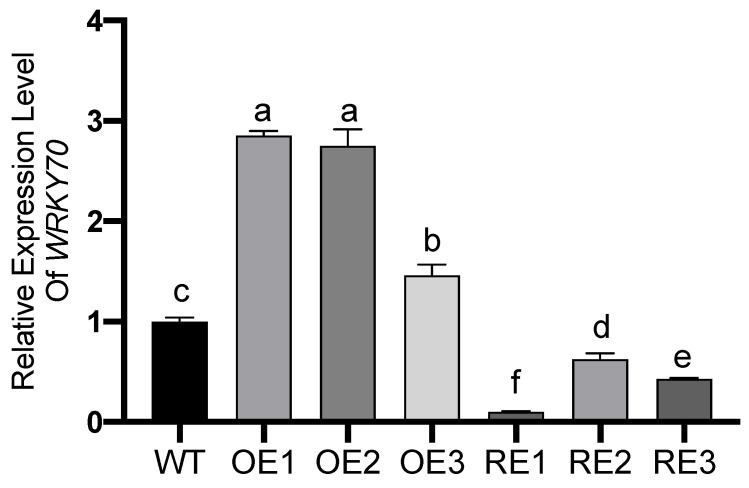
The expression levels of *PsnWRKY70* in leaves of transgenic and WT lines in poplar. The means and standard errors were calculated with six replications. Multiple comparison was used via Duncan test. Different letters indicate significant differences (*p* < 0.05).

The *PsnWRKY70* expression levels of OE1, OE2, and OE3 were significantly up regulated compared with the WT lines. On the contrary, the *PsnWRKY70* gene was significantly down-regulated in the interference expression lines RE1, RE2 and RE3. Among them, the expression level of OE1 line was the highest, which was 2.85 times that of WT; while the expression level of RE1 line was the lowest, which was 0.10 of that of WT (Figure 2).

### 2.2. Growth Performance of Transgenic Lines under NaHCO_3_ Stress

**Figure 3:**
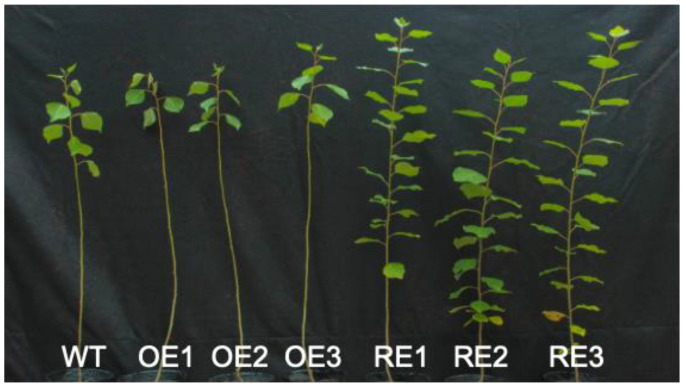
Phenotypic images among poplar transgenic and WT lines at day 15 under NaHCO_3_ stress treatment.

Under NaHCO_3_ stress, the tested lines showed different degrees of damage (Figure 3). On day 15, the leaves of the WT line and the *PsnWRKY70* OE lines had more chlorosis and yellowing, and some leaves had fallen off; the alkali damage was serious. However, the *PsnWRKY70* RE lines only saw a few leaves fall off, and alkali damage was slight.

The plant height at day 15 under NaHCO_3_ stress was investigated, and the net growth and relative height growth indexes were calculated. The results showed that there was no significant difference in plant height among the tested lines before NaHCO_3_ stress treatment. After NaHCO_3_ stress treatment, the net growth and relative growth of RE lines were not significantly different from those of WT; however, the net growth and relative growth of OE lines were significantly lower than those of WT (*p* < 0.05). The average net growth of OE lines was 0.64 times that of WT. The relative high growth of the OE3 line was only 0.48 (Table 1). Under NaHCO_3_ stress, the salt damage of RE lines had less of an effect on plant growth, while there was a more severe degree of salt damage for OE lines. The net growth rate was the slowest.

### 2.3. Alkali Damage Index

**Figure 4:**
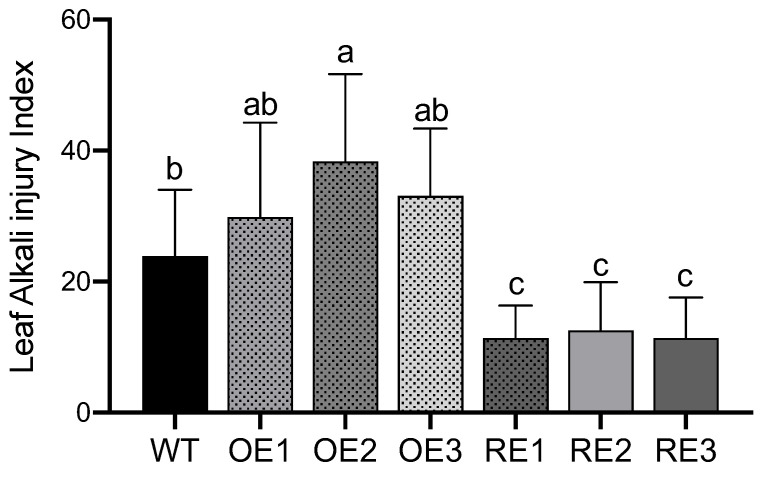
The difference of leaf alkali injury indexes among poplar transgenic and WT lines under NaHCO_3_ stress treatment. The means and standard errors were calculated with six replications (*p* < 0.05). Multiple comparison was used via Duncan test. Different letters indicate significant differences.

The alkali damage index of the tested lines was investigated at day 15 under NaHCO_3_ stress. The results showed that the alkali damage index of the RE line was lower than that of the WT and OE lines. The average alkali damage index of the RE lines was 11.77%, while the alkali damage indices of the WT and OE lines were as high as 23.90% and 33.78%. Among them, the alkali damage index of the RE1 lines was the lowest, which was only 11.37%, while the alkali damage index of OE2 was as high as 38.36% (Figure 4).

### 2.4. Net Photosynthetic Rate under NaHCO_3_ Stress

**Figure 5:**
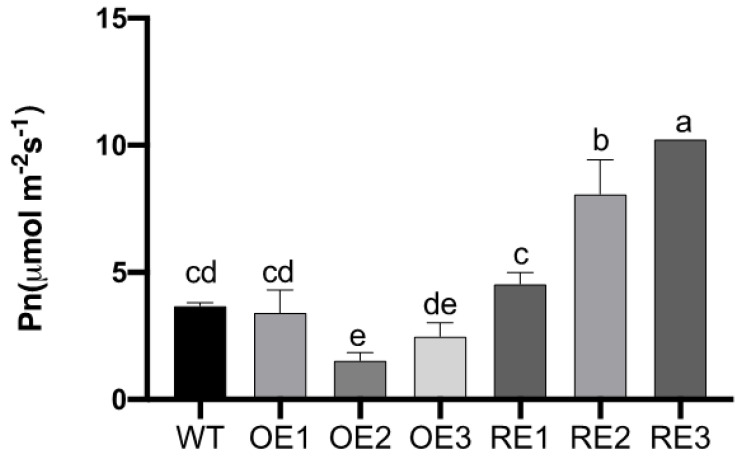
The difference of net photosynthesis rate among poplar transgenic and WT leaves under NaHCO_3_ stress treatment. The means and standard errors were calculated with three replications (*p* < 0.05). Multiple comparison was used via Duncan test. Different letters indicate significant differences.

The net photosynthetic rate (Pn) of leaves at day 15 under NaHCO_3_ stress was determined. The Pn of the RE lines was significantly higher than that of the WT and OE lines under NaHCO_3_ stress at day 15. The mean was 3.74 and 5.01 times higher than that of the WT and OE lines, respectively. The Pn of RE3 lines can reach 10.23 µmol m^−2^s^−1^. However, the Pn of the OE lines was lower than that of the WT. OE2 had significantly lower than WT. This indicated that the net photosynthetic rate of WT and OE lines (especially OE2) was seriously affected by alkali stress, while the net photosynthetic rate of the RE line was less affected by alkali stress (Figure 5).

### 2.5. Physiological Parameters

**Figure 6:**
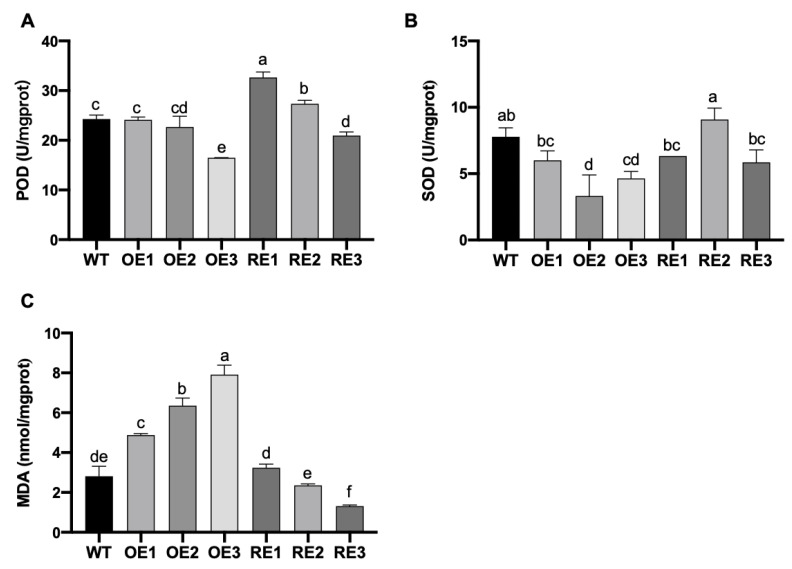
The difference of physiological indexes among poplar transgenic lines under NaHCO_3_ stress. (**A**) SOD activity of transgenic lines. (**B**) SOD activity of transgenic lines. (**C**) MDA content of different transgenic lines. The means and standard errors were calculated with three replications (*p* < 0.05). Multiple comparison was used via Duncan test. Different letters indicate significant differences.

SOD and POD activities were measured in each transgenic line of poplar. After NaHCO_3_ stress at day 15, POD activity was significantly higher in the RE line than in WT, while there was no significant difference in OE lines (Figure 6A). The SOD activity of the OE lines was significantly lower than WT, while there was no significant difference between RE lines and WT (Figure 6B). These results suggest that *PsnWRKY70* negatively regulates the activities of SOD and POD under NaHCO_3_ stress and ultimately cause the accumulation of ROS in plants. Figure 6C shows the MDA content of the transgenic lines at day 15 under NaHCO_3_ stress. The MDA content of the OE lines was significantly higher than that of WT. The MDA content of the RE lines was lower than that of WT. The MDA content of RE3 was significantly lower than that of WT (*p* < 0.05). The results showed that the low expression of the *PsnWRKY70* gene could reduce the accumulation of MDA and stabilize the cell membrane structure.

### 2.6. Transcriptome Analysis

**Figure 7:**
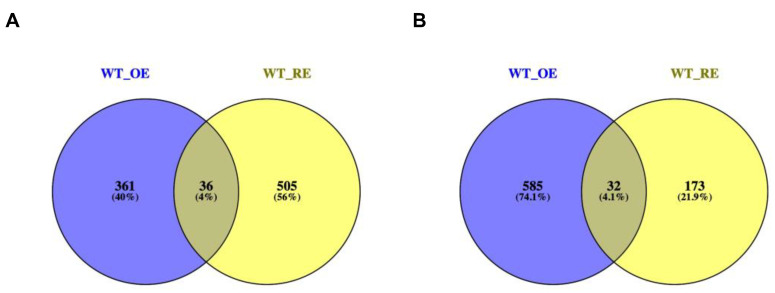
A Venn diagram of the number of differentially expressed genes. (**A**) Upregulated DEGs between OE and RE. (**B**) Downregulated DEGs between OE and RE.

To analyze changes in gene expression patterns, RNA-seq was performed using the fifth leaf of WT, OE1, and RE1 at day 15 under NaHCO_3_ stress. The thresholds of DEGs between OE vs. WT and RE vs. WT were used with a fold change ≥2 and a *p*-value < 0.05. The results showed that there were 361 and 505 exclusively upregulated DEGs in the OE and RE lines. There were 36 overlap DEGs between OE and RE (Figure 7A). There were 585 and 173 exclusively downregulated DEGs in the OE and RE lines. There were 32 overlap DEGs between OE and RE (Figure 7B).

**Figure 8:**
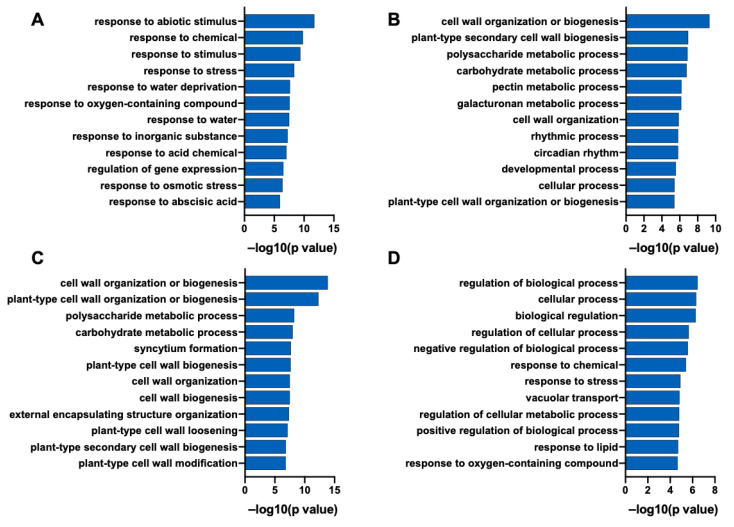
A GO enrichment analysis of DEGs in *PsnWRKY70* transgenic poplar. (**A**) A GO enrichment analysis of uniquely upregulated genes in OE lines. (**B**) A GO enrichment analysis of uniquely downregulated genes in OE lines. (**C**) A GO enrichment analysis of uniquely upregulated genes in RE lines. (**D**) A GO enrichment analysis of uniquely downregulated genes in RE lines.

To further analyze the molecular function of the *PsnWRKY70* gene, GO enrichment analysis was performed based on identified DEGs (Figure 8). GO analysis showed that the 361 upregulated DEGs in OE lines were enriched in response to abiotic stimulus, in response to chemical, and in response to stimulus (Figure 8A). The 505 upregulated DEGs in RE lines were enriched in cell wall organization or biogenesis, the polysaccharide metabolic process, and the carbohydrate metabolic process (Figure 8C); GO analysis showed that the 585 downregulated DEGs in OE were enriched in cell wall organization or biogenesis, the polysaccharide metabolic process, and the carbohydrate metabolic process (Figure 8B). The 173 downregulated DEGs in RE were enriched in the regulation of the biological process and cellular process (Figure 8D). This suggests that the *PsnWRKY70* regulation of biological processes plays an important role in response to NaHCO_3_ stress in poplar.

### 2.7. Expression of Genes Related to Cell Wall Tissue Biogenesis and Polysaccharide Metabolism

**Figure 9:**
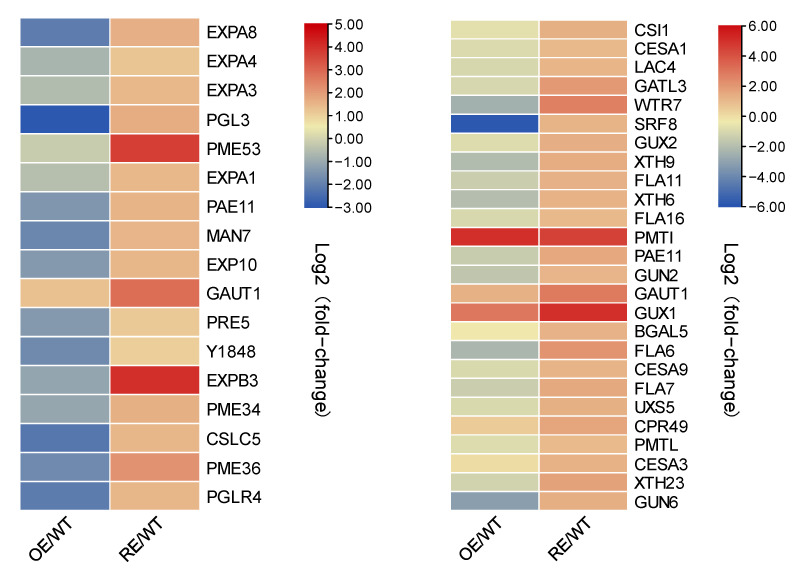
The expression of genes related to cell wall tissue biogenesis and polysaccharide metabolism in poplar.

*PsnWRKY70* transgenic RE lines showed their tolerance to NaHCO_3_ stress. Up-regulated genes of RE lines were enriched in the cell wall organization and biogenesis pathway (Figure 9). The cell wall organization includes expansions (expansion proteins are cell wall relaxing proteins with non-hydrolytic activity) *EXPA8*, *EXPA4*, *EXPA3*, *EXPA1*, *EXPB3*, *EXP10*; At the same time, pectin methylesterase *PME53*, *PME34*, *ME36*; *XTH9*, *XTH6*, *XTH23*; *CESA1*, *CESA3*, *CES9*; *FLA11*, *FLA16*, *FLA7* and other genes were significantly up-regulated in RE lines. These results suggest that the changes in cell wall organization or biogenesis-related gene expression in RE lines play a crucial role in NaHCO_3_ tolerance.

## 3. Discussion

The WRKY family is one of the largest families of transcription factors and plays a crucial role in abiotic stress. Previous studies showed that different members of the WRKY family play different roles in response to abiotic stresses [24,25,26,27]. In *Eriobotrya japonica*, *EjWRKY17* enhances drought resistance by reducing reactive oxygen species levels, closing stomata, and increasing the expression of drought resistance-related genes [24]. Overexpression of *TaWRKY46* enhances osmotic tolerance by inducing the expression of some stress-related genes (*P5CS1*, *RD29B*, *DREB2A*, *ABRE*, *CBF2*, *CBF3*) in transgenic *Arabidopsis* [28]. Overexpression of *GhWRKY1*-like increases the drought tolerance of cotton by promoting ABA synthesis in *Gossypium hirsutum* [12]. However, some WRKY genes play a negative role in the plant response to abiotic stress. For example, overexpression of *CaWRKY27* leads to decreased expression of most reactive oxygen species scavenging genes and ABA synthesis genes, which can make plants more sensitive to salinity and osmotic stress [29]. The down-regulated expression of *GhWRKY6* and *GhWRKY27a* genes in cotton can increase the salinity and drought tolerance of plants [30]. The *PalWRKY77* transcription factor negatively regulates salt tolerance in poplar. *PalWRKY77* binds to the promoters of ABA and salt-induced genes *PalNAC002* and *PalRD26* to negatively regulate poplar abscisic acid signaling [31]. In this study, we showed that RE lines could enhance the antioxidant capacity of plants. RE lines suffered less membrane damage and less stress damage. Our results indicated that *PsnWRKY70* plays a negative regulatory role in response to NaHCO_3_ stress. *PsnWRKY70* gene interference expression RE lines enhanced the tolerance of plants to NaHCO_3_.

Expansin is a cell-wall-loosening protein known to disrupt hydrogen bonds between xyloglucan and cellulose microfibrils. The expression of expansin is increased in plants under various abiotic stresses and plays an important role in adaptation to these stresses [32]. Ectopic expression of wheat expansin gene *TaEXPA2* improves the salt tolerance of transgenic tobacco (*Nicotiana tabacum*) by regulating Na^+^/K^+^ and antioxidant capacity [33]. Rice *OsEXPA7* plays an important role in enhancing salt stress tolerance by coordinating sodium transport, ROS scavenging and cell wall loosening [34]. Our research shows that *PsnWRKY70* negatively regulates the activities of SOD and POD under NaHCO_3_ stress, ultimately causing the accumulation of ROS in plants. This may be related to the significant upregulation of our cell expansion proteins *EXPA8*, *EXPA4*, *EXPA3*, *EXPA1*, *EXPB3* and *EXP10* in the RE lines. XTH Xyloglucan endotransglucosylase/hydrolase (XTH) family enzymes play an important role in the restructuring of cellulose microfibril load-bearing cross-links [35]. XTH can be involved in plant responses to abiotic stress by remodeling the cell wall. In pepper (*Capsicum annuum*), *CaXTH1*, *CaXTH2* and *CaXTH3* genes were up-regulated under drought, high salt and low temperature conditions and overexpression of *CaXTH3* enhanced the tolerance of *Arabidopsis* and tomato to salt and drought stress [36,37]. XTH gene palisade tissue cells are highly dense and the intercellular space between mesophyll cells is reduced. In addition to the NaCl dilution effect, these anatomical changes increased the water-holding capacity of leaves, thereby reducing the salt concentration of fleshy tissues and mesophyll cells. *PeXTHK* gene in *Populus euphratica* can increase tolerance to salt stress in *Arabidopsis* [38]. In general, the XTH gene can improve the water holding capacity of the plant, thereby enhancing the salt tolerance of the plant. This is consistent with our experimental results that the upregulation of *XTH9*, *XTH6* and *XTH23* in RE lines may also lead to the water retention ability of plants and enhance the tolerance of poplar to NaHCO_3_. Transcriptome GO enrichment analysis found that the differential genes up-regulated in RE lines were significantly enriched in the cell wall organization or biogenesis process (Q-value < 0.05). Cell expansion proteins *EXPA8*, *EXPA4*, *EXPA3*, *EXPA1*, *EXPB3*, *EXP10*, *PME53*, *PME34*, *PME36*, *XTH9*, *XTH6*, *XTH23*, *CESA1*, *CESA3*, *CES9*, *FLA11*, *FLA16*, *FLA7* genes were significantly up-regulated in RE lines. These results indicated that the changes of gene expression levels related to cell wall organization or the biogenesis process in RE lines played a crucial role in NaHCO_3_ stress tolerance. Therefore, *PsnWRKY70* may regulate the expression of cell wall-related genes and enhance the salt-tolerance of plants under NaHCO_3_ stress. Our study provided novel insights into the molecular mechanisms underlying *PsnWRKY70* regulation of the saline-alkali tolerance in poplar.

## 4. Materials and Methods

### 4.1. Plant Materials

Six *PsnWRKY70* poplar (*Populus simonii × Populus nigra*) transgenic lines developed from our previous study [23] were used in this study. There were *PsnWRKY70* overexpression (OE) lines OE1, OE2, OE3 and gene interference expression (RE) lines RE1, RE2, RE3. Wild type (WT) was used as control. Cuttings were collected from 2-year-old transgenic plants and planted in 30 × 36 cm pots in a greenhouse. The substrate was peat soil: river sand: black soil (v/v) = 2:2:1. Three-month-old seedlings were treated with NaHCO_3_ solution with a concentration of 200 mmol/L for 15 days, and seven plants of each line were irrigated with water. After 15 days, the NaHCO_3_ solution treatment was ended, and the follow-up routine management was adopted.

### 4.2. Molecular Validation of Transgenic Lines

The genomic DNA of poplar leaves was extracted with a DNAquick Plant System (TIANGEN BIOTECH, Beijing, China), and *PsnWRKY70*-F: 5′CACCATGGATTCTTCTTGGCATGGGAAT3′& *PsnWRKY70*-R: 5′AAATCCGAAAACACCATCATCATCG3′ were used as upstream and downstream primers for OE detection of overexpressed lines; PCR detection of RE lines was carried out with specific primers pRS300-F: 5′-CTGCAAGGCGATTAAGTTGGGTAAC-3′& pRS300-R: 5′-GCGGATAACAATTTCACACAGGAAACAG-3′ for RNAi expression vector; nptII-F: 5′-GGTGGAGAGGCTATTCGGCTATGA-3′& nptII-R: 5′-TGATATTCGGCAAGCAGGCATCG-3′ was used as a primer to carry out PCR detection on OE, RE and WT lines. Polymerase chain reaction (PCR) was performed in a 20 μL volume consisting of 10 μL 1.1 × T3 Super PCR Mix (Tsingke Biological Technology, China), 2 μL 10 10 μmolL^–1^ of primer, 7 μL deionized water, and 1 μL DNA template. The PCR program was run at 94 °C for 3 min for pre-denaturation, followed by 30 cycles (1 min at 94 °C, 1 min at 58 °C, and 20 s at 72 °C), and finally extended at 72 °C for 5 min. The amplified DNA fragments were electrophoresed on a 1% agarose gel.

### 4.3. PsnWRKY70 Gene Expression

Total RNA was extracted from leaves of OE, RE and WT lines using Universal Plant Total RNA Extraction Kit (Spin-column) I (BioTeke Corporation, Beijing, China). Double-stranded cDNA was synthesized separately with Toyobo Reverse Transcription Kit ReverTra Ace^®^ qPCR RT Master Mix with gDNA Remover (Toyobo, Osaka, Japan). With poplar leaf cDNA as the template, *PsnWRKY70*-F: 5′-GGTAAGGACAGGAGAGGAT-3′, *PsnWRKY70*-R: 5′-CGTGATATGTTGTGCGGTAT-3′ as primers, we used a Toyobo SYBR^®^ Green Real-time PCR Master Mix Plus in ABI. qRT-PCR was performed on an ABI-7500 quantitative PCR instrument. The relative expression was calculated by 2(−∆∆CT). The internal reference gene is 18S rRNA.

### 4.4. Plant Height and Leaf Alkali Damage Index

On the 15th day after the NaHCO_3_ stress treatment, plant heights were measured with a tower ruler, and their relative growth was calculated. During the stress process, the phenotypic changes of the plants were continually observed and photographed. The alkali damage index of the leaves of each line under NaHCO_3_ stress was counted on the 15th day of stress. Regarding the alkali damage grading standard system: grade 0, leaves were healthy; grade 1—yellowing areas were less than 20% of the total leaf area; grade 2—yellowing occurred in 50% of the total leaf area; grade 3—more than 80% of the total leaf areas appeared to be yellowing; grade 4—leaves died. Calculation of the leaf alkali damage index: LSI = [∑(si × Nsi) ∕(NsT × Gsmax)] × 100%. Si: different alkali damage grades (0~4), Nsi: the number of si grade alkali damage leaves per seedling, NsT: the total number of leaves per seedling, Gsmax: the highest alkali damage grade.

### 4.5. Physiological and Photosynthetic Parameters

The net photosynthetic rates of WT, OE and RE were measured using a Li-6400 portable photosynthesis instrument at 9:00–11:00 a.m. on the 15th day after stress. Three plants were measured for each line. The light intensity was set to 1000 μmoL·m^−2^·s^−1^, and the CO_2_ concentration was set to 400 μmoL·m^−2^·s^−1^. SOD activity, POD activity, and MDA content in leaves were determined using a Malondialdehyde Detection Kit (Nanjing Jiancheng Bioengineering Institute, Nanjing, China).

### 4.6. RNA-Seq Data Analysis

Total RNA was extracted from the whole leaves of WT, OE and RE transgenic plants using a Universal Plant Total RNA Extraction kit (BioTeke Corporation, Beijing, China) after NaHCO_3_ stress for 15 days. A NanoDrop 2000 was used to evaluate RNA purity and integrity. RNA samples were submitted to the Illumina X10 platform for high-throughput sequencing. The sequencing read length was 150 bp paired end reads. The original sequence reads were filtered to obtain high-quality clean reads before subsequent analysis. Clean reads were mapped to the *Populus trichocarpa* genome using hisat2 [39]. Stringtie software was used to count reads mapped to the genome [40]. DEseq2 was used for the significance analysis of differentially expressed genes (DEGs) (*p* < 0.05, Fold Change ≥2) [41]. DEGs were used for a gene ontology (GO) enrichment analysis using the GO enrichment tool (http://geneontology.org, accessed on 24 February 2021) with a *p*-value of less than 0.01 as the threshold for significant enrichment [42,43,44].

### 4.7. Statistical Analysis

All experiments were performed with three biological replicates. The mean was shown as the mean ± standard deviation (SD). The data were analyzed via one-way ANOVA using Duncan’s multiple range test (*p* < 0.05) in SPSS version 24.0.

## Figures and Tables

**Table 1:** The difference of plant height growth among OE, RE and WT lines under NaHCO_3_ stress treatment.

Lines	Plant Height (cm)		Net Growth (cm)	Relative Growth
	Stress Day 0	Stress Day 15		
WT	60.71 ± 7.52 c	112.00 ± 10.21 a	51.29 ± 11.01 a	0.86 ± 0.23 a
OE1	72.67 ± 10.23 abc	114.17 ± 10.76 a	41.50 ± 6.69 bc	0.58 ± 0.14 bc
OE2	79.33 ± 8.82 a	116.50 ± 2.88 a	37.17 ± 9.52 c	0.48 ± 0.17 c
OE3	69.17 ± 8.28 abc	110.33 ± 12.75 a	41.17 ± 6.94 bc	0.60 ± 0.10 bc
RE1	68.50 ± 11.96 abc	114.83 ± 7.00 a	46.33 ± 6.15 abc	0.71 ± 0.25 abc
RE2	73.75 ± 12.89 ab	119.13 ± 8.36 a	45.38 ± 6.63 abc	0.65 ± 0.24 abc
RE3	66.25 ± 9.10 bc	116.13 ± 8.71 a	49.88 ± 6.64 ab	0.77 ± 0.20 ab

Net growth (cm) was the difference between plant height (cm) at 15 days and 0 days under NaHCO_3_ stress treatment. Relative growth was the ratio of net growth (cm) and plant height (cm) at 0 day. Data indicate means ± STDEV (n = 10, *p* < 0.05). Multiple comparison was used by Duncan test. Different letters indicate significant differences.

## Data Availability

All RNA-seq data have been archived in the NCBI Sequence Read Archive (SRA), accession number PRJNA881620.
